# Comparison of the efficacy of two hand decontamination methods: Alcoholic septicidine and povidone–iodine solutions

**DOI:** 10.1371/journal.pone.0336437

**Published:** 2025-11-21

**Authors:** Nooshin Bazzazi, Hamidreza Ghasemi Basir, Roya Najafi Vosough, Fatemeh Eslami, Negar Akbarzadeh

**Affiliations:** 1 Department of Ophthalmology, School of Medicine, Hamadan University of Medical Sciences, Hamadan, Iran; 2 Department of Pathology, School of Medicine, Hamadan University of Medical Sciences, Hamadan, Iran; 3 Vice-Chancellor for Research and Technology, Hamadan University of Medical Sciences, Hamadan, Iran; 4 Student Research Committee, Hamadan University of Medical Sciences, Hamadan, Iran; Democritus University of Thrace, GREECE

## Abstract

**Background and aim:**

Surgical site infections (SSIs) are a critical concern in ophthalmic surgical settings, where rigorous hand hygiene is essential to prevent postoperative complications. This study aimed to compare the efficacy of two antiseptic agents, Septicidine and Betadine, in reducing bacterial contamination on the hands of surgical staff.

**Methods:**

Thirty ophthalmic surgeons and operating room personnel participated in the study. The participants scrubbed their hands with either Septicidine (alcohol-based antiseptic) or Betadine (iodine-based antiseptic). Bacterial cultures were obtained before and after hand scrubbing. The presence of bacteria, including antibiotic-resistant strains, was assessed and compared between the two groups over a four-month period at Sina Hospital in Hamadan.

**Results:**

A total of 120 hand culture samples were collected. Both antiseptics significantly reduced bacterial contamination. However, residual bacterial growth was observed in 8 out of 120 Septicidine samples and 10 out of 120 Betadine samples. Although Septicidine showed a slightly lower rate of residual contamination, the difference was not statistically significant (P = 0.624). The most commonly isolated organisms were Staphylococcus species, some of which exhibited resistance to antibiotics such as cotrimoxazole, clindamycin, and vancomycin.

**Conclusion:**

Both Septicidine and Betadine were effective in reducing hand bacterial load, although neither achieved complete sterilization. Alcohol-based solutions such as Septicidine may offer benefits in terms of ease of use and skin compatibility. Improving iodine-based solutions such as Betadine application techniques may enhance its efficacy. Further research is warranted to optimize hand hygiene protocols in surgical environments.

## Introduction

Surgical site infections (SSIs) are major causes of postoperative morbidity across all types of surgery, but they are particularly critical in ophthalmic procedures due to the risk of severe complications such as endophthalmitis. Although rare, postoperative endophthalmitis is a serious and vision-threatening complication of intraocular surgery. Common sources of infection include the patient’s eyelid and conjunctival flora, contaminated instruments or solutions, and transmission from surgeons or other operating room personnel [1,2]. Preventing such infections is therefore essential for ensuring successful surgical outcomes and protecting patients’ vision. The hands of surgical staff represent one of the most significant sources of microbial contamination in the operating room. Despite the routine use of sterile gloves, the risk of contamination remains, especially in cases of glove perforation or inadequate hand hygiene practices [3,4]. As such, effective preoperative hand scrubbing is a critical component of surgical infection control protocols. Since the transfer of microorganisms from the hands of the surgical team is a key factor in the development of SSIs, the primary goal is to reduce the microbial load on the skin to the lowest possible level, thereby minimizing the risk of introducing pathogens into the surgical site [5].

Various antiseptic agents have been developed and used over the years to achieve this goal. Traditional surgical scrubbing is most commonly performed with povidone-iodine or chlorhexidine gluconate. Povidone-iodine, often marketed under the trade name Betadine, remains one of the most widely used antiseptics in surgical settings [6–8]. It is a broad-spectrum antimicrobial agent that is effective against a wide range of bacteria, viruses, and fungi [9,10]. Povidone-iodine exerts its antimicrobial effect by releasing free iodine, which rapidly penetrates microbial cell walls and inactivates essential proteins and nucleic acids through oxidation, ultimately leading to microbial cell death [11]. Its long history of use and well-established efficacy have made it a standard choice in many hospitals worldwide [12]. On the other hand, Alcoholic Septicidine Solution is a more recent antiseptic agent, recognized for its rapid action and broad-spectrum antimicrobial activity. As an alcohol-based sanitizer, it effectively denatures microbial proteins [13,14]. The primary mechanism of action of alcohols, such as ethanol and isopropanol, is the denaturation of proteins, which quickly kills microorganisms [15]. Alcoholic solutions are highly favored in time-sensitive settings, such as surgical preparation, due to their rapid disinfection capabilities. Their quick evaporation eliminates the need for water and leaves the skin dry, ensuring immediate readiness for subsequent procedures [16].

While Povidone-Iodine and Alcoholic Septicidine Solution are both widely used, their relative effectiveness remains a subject of ongoing debate within the medical community, especially concerning their application in diverse surgical contexts [8,17]. Some studies suggest that alcohol-based solutions may be more effective in certain scenarios due to their immediate action and ease of use, while others highlight the persistent antimicrobial activity of iodine-based solutions [18]. In ophthalmic surgery, where precision and the prevention of infection are paramount, the choice of antiseptic for hand scrubbing can have significant implications [3]. Despite the widespread use of Povidone-Iodine and Alcoholic Septicidine solutions, there is a lack of comprehensive studies specifically comparing their effectiveness in eye surgery. This study aims to address this gap by directly evaluating how well each antiseptic reduces microbial contamination on the hands of surgical staff prior to ophthalmic procedures. The results will not only enhance the current knowledge base but also offer practical guidance for surgical teams in choosing the most appropriate preoperative hand antiseptic. Through systematic assessment of microbial load reduction, this research intends to inform best practices in ophthalmic surgery and potentially influence hospital protocols to improve patient safety and surgical outcomes.

## Materials and methods

### Study design and setting

This comparative study was conducted at Sina Hospital, Hamadan, Iran, over a four – month’s period from 2022–2023. This study aimed to assess the effectiveness of two preoperative hand decontamination methods: Povidone-Iodine, Betadine 7.5% (Mehriz Industrial, Kavir Yazd) and Alcoholic Septicidine (Ethanol 70% -Chiorhexidine 0.1%, Behnam Shimi – Iran) solutions. The research involved surgeons and operating room staff who regularly participated in ophthalmic surgeries.

### Participants

Lindström et al. evaluated two methods of hand disinfection: Sterillium and Hibiscrub. According to their findings, the mean (SD) number of colony-forming units (CFUs) was 2.01 (0.98) for Sterillium and 1.45 (0.50) for Hibiscrub [19]. Based on these results, a sample size of 30 participants was determined to be sufficient to detect a statistically significant difference at the 95% confidence level and 80% statistical power.

A total of 30 participants were enrolled in the study, including 15 surgeons and 15 operating room staff members. All participants were required to meet the inclusion criteria, which included having no known allergies to the antiseptics used, and not having used systemic antibiotics within two weeks prior to the study. The participants were fully informed about the study’s objectives and provided written consent. Additionally, this study was approved by the ethics committee of the hospital.

### Hand scrubbing protocol

The study was divided into two phases. During the first week, the participants used Alcoholic Septicidine Solution for preoperative hand scrubbing, following the hospital’s standard hand hygiene protocol. This involved scrubbing for at least three minutes, ensuring thorough coverage of all hand and forearm surfaces which were then left to dry. In the second week, the same participants used Povidone-Iodine (Betadine) following an identical scrubbing protocol. This protocol was repeated over a four-month period. After the hand samples were taken and before surgery each personnel once again disinfected their hands using routine scrub method.

### Sample collection and bacterial culture

Bacterial samples were collected from the participants’ hands immediately before and after scrubbing during both weeks of the study in each period. Sterile cotton swabs were used to collect the samples from the palm, fingertips, and between the fingers. These swabs were then inoculated onto nutrient agar plates and incubated at 37°C for 48 hours. The bacterial colonies that grew on the plates were counted, and the microbial load before and after hand scrubbing was recorded. Further microbiological analyses were performed on the positive post-scrubbing samples to identify the bacterial species present. Bacterial isolates were subcultured and identified using standard microbiological techniques, based on gram staining results, specific biochemical tests were selected to ensure accurate classification. For gram-negative bacilli, identification was performed using Citrate utilization, Oxidation-Fermentation (OF) test, Sulfide-Indole-Motility (SIM), Urease test, Methyl Red-Voges Proskauer (MRVP), Phenylalanine deaminase, Lysine decarboxylase, ONPG were utilized. For gram-positive cocci, particularly *Staphlococcus* species, Catalase and Tube Coagulase tests were initially performed to differentiate between *Staphylococcus aureus* coagulase-negative Staphylococci. DNase agar, and Mannitol salt agar, Bile Esculin hydrolysis were also used for further identification. Antibiotic susceptibility testing was carried out using the Kirby-Bauer disk diffusion method on Mueller-Hinton agar plates, and the results were interpreted in accordance with the Clinical and Laboratory Standards Institute (CLSI) guidelines.

### Staff preference assessment

At the end of the study, each participant was asked a single direct question regarding their preference between the two antiseptic solutions (Alcoholic Septicidine vs. Betadine), based on their overall experience. Their responses were recorded for descriptive comparison.

### Data analysis

The data collected were analyzed using descriptive statistics to summarize the reduction in microbial load achieved by each antiseptic. The effectiveness of Povidone-Iodine and Septicidine was compared using McNemar’s test, a statistical method appropriate for paired nominal data. A p-value of less than 0.05 was considered statistically significant.

## Results

A total of 30 individuals from four periods (120 cases) participated in the study and were sampled after each disinfection method. The presence of bacteria was assessed both before and after hand rub before surgery, with the results revealed that bacterial cultures were positive in 8 cases after using Septicidine and in 10 cases after using Betadine. The differences in positive bacterial cultures between the two solutions were not statistically significant (P = 0.624), as shown in Table 1.

**Table 1:** Comparison of positive bacterial cultures between septicidine and betadine solutions.

Bacterial Cultures	Septicidine	Betadine	Total	P-value
Positive	8	10	18	0.624
Negative	112	110	222	
Total	120	120	240	

This study further analyzed the types of microbial flora remaining on the hands of the participants. Most of the bacteria isolated after hand scrubbing with Septicidine were different species of Staphylococcus, with Staphylococcus aureus being detected in 2 cases and other Staphylococcus species in 5 cases. Table 2 shows the distribution of microbial flora after using Septicidine. Similarly, after hand scrubbing with Betadine, most of the isolated bacteria were Staphylococcus species, with Staphylococcus aureus found in 4 cases and other Staphylococci in 5 cases (Table 2). We also compared the effectiveness of both antiseptic solutions in removing the microbial flora from the hands of the participants. The findings showed no significant difference in the presence of bacteria on the hands of individuals after using either Septicidine or Betadine, as shown in Table 2.

**Table 2:** Distribution and comparison of microbial flora after hand scrubbing with septicidine and betadine.

Type of microbial flora	Septicidine	Betadine	P-value
Staphylococcus aureus	2	4	0.877
Staphylococcus Spp	5	5	
Diphtheroid	1	1	
Negative	112	110	
Total	120	120	

The antibiotic susceptibility of the isolated bacterial species was also evaluated. The results for the Septicidine-treated group are presented in Table 3, showing high sensitivity to most antibiotics, with no resistance observed against Amikacin, Ciprofloxacin, and Vancomycin. Similar to the Septicidine group, high sensitivity was observed, although some resistance was noted, particularly against Cefazolin and Clindamycin. Further comparisons were made between the antibiotic susceptibility profiles for both antiseptic solutions against specific antibiotics. the results revealed that there was no statistically significant difference in antibiotic resistance between the two groups across most antibiotics tested (*P* > 0.05).

**Table 3:** Antibiotic susceptibility of microbial flora after hand scrubbing with septicidine and betadine.

Antibiotic	Septicidine		Betadine		P-value
	Sensitive (number = 8)	Resistant (number = 8)	Sensitive (number = 10)	Resistant (number = 10)	
Amikacin	8	0	10	0	–
Cefazolin	7	1	8	2	0.671
Ceftriaxone	7	1	9	1	0.867
Ciprofloxacin	8	0	10	0	–
Doxycycline	7	1	10	0	0.250
Cotrimoxazole	6	2	6	4	0.502
Clindamycin	6	2	7	3	0.814
Vancomycin	8	0	7	3	0.090

Additionally, in the present study we have surveyed the participants’ preferences for using either Betadine or Septicidine for hand scrubbing before eye surgery. The results, presented in Table 4, indicated that the majority preferred Septicidine over Betadine.

**Table 4:** Staff preferences for betadine and septicidine for hand scrubbing before eye surgery.

Antiseptic Solution	Number	Percentage
Betadine	8	26.7%
Septicidine	22	73.3%
Total	30	100%

## Discussion

This study aimed to evaluate and compare the efficacy of two antiseptic solutions, Alcoholic Septicidine and Povidine-Iodine (Betadine), in reducing bacterial contamination on the hands of ophthalmic surgeons and operating room personnel. Effective hand hygiene is crucial in preventing surgical site infections (SSIs), making the choice of antiseptic solutions critical in clinical settings. Some of the surgeons routinely use Betadine because it is widely available, inexpensive and has a wide spectrum of acting against novel pathogens. Others prefer Septicidine because of its rapid action and ease of use.

Our findings demonstrate that while both Septicidine and Betadine are effective in reducing microbial flora, neither solution completely eliminated bacterial presence on the hands of surgical staff. Positive bacterial cultures suggest that neither antiseptic agant was able to completely eradicate bacteria. Specifically, post-scrub bacterial presence was found in 6.67% of cases with Septicidine and8.33% with Betadine, though the difference was not statistically significant. These results are consistent with studies by Pegou et al. and Tanner et al., which also reported limited efficacy of traditional scrubbing methods in fully reducing bacterial counts on the hands of operating room personnel [20,21]. The predominant bacteria remaining after scrubbing were Staphylococcus species, particularly Staphylococcus aureus. The persistence of these bacteria, even after rigorous hand scrubbing, raises concerns about the risk of post-surgical infections. While Betadine was slightly less effective in reducing the presence of S. aureus compared to Septicidine, this difference was not significant, suggesting that both antiseptics perform similarly in clinical practice.

In our study, we observed a trend toward slightly higher antibiotic resistance in samples treated with Betadine, particularly against Cotrimoxazole, Clindamycin, and Vancomycin (Table 3). One possible explanation for this observation is the use of non-sterile water for rinsing after Betadine application, which may have reintroduced resistant bacteria and affected the overall antiseptic efficacy. This hypothesis is consistent with the findings of Ghods and Irajian [22], who reported no significant advantage of Betadine over alcohol-based scrubbing in eliminating bacterial colonies. However, it is important to note that the observed differences in resistance rates were not statistically significant, and our data do not provide definitive evidence to establish a causal link between the use of non-sterile water and increased antibiotic resistance. Further studies with controlled rinsing protocols and larger sample sizes are needed to clarify this potential association.

The study also explored the antibiotic susceptibility of the residual bacterial flora. Most of the bacteria were sensitive to common antibiotics, such as Amikacin and Ciprofloxacin. However, there was notable resistance to Cotrimoxazole, Clindamycin, and Vancomycin, particularly after the use of Betadine. This could be attributed to the fact that hand scrubbing with Betadine, followed by rinsing with nonsterile water, might be less effective in eliminating resistant bacterial strains. Previous studies, such as those by Ghods and Irajian, have also shown no significant difference in the efficacy of Betadine compared with alcohol-based scrubbing in eliminating bacterial colonies [22]. The results of our study align with other research suggesting that alcohol-based antiseptic solutions offer several advantages over traditional Betadine scrubbing. For example, alcohol-based scrubs do not require water, which reduces the risk of contamination from non-sterile water sources and saves time. Additionally, these solutions have been shown to be more cost-effective and have a longer-lasting antimicrobial effect, as reported by Gancalves et al. [23]. Alcoholic Septicidine Solution demonstrated a marginally higher efficacy in reducing the overall microbial load, particularly in completely eliminating Staphylococcus species from all post-scrubbing samples. This outcome is consistent with the rapid and potent bactericidal action of alcohol-based solutions, which are known to denature proteins and disrupt cell membranes effectively. The rapid evaporation of alcohol also allows for quicker surgical preparation, which can be advantageous in high-volume surgical settings [24–26]. Moreover, studies by Lai et al. [25] and Ali et al. [27] have demonstrated that alcohol-based solutions are preferred for their faster action and better skin tolerance, which might explain the higher preference for Septicidine among the participants in our study.

Based on our findings and corroborating evidence from other studies, it is recommended that alcohol-based solutions like Septicidine be considered as a preferred method for hand scrubbing in operating rooms, especially in ophthalmic surgeries where precision and sterility are paramount. However, for Betadine, improvements in scrubbing techniques and the use of sterile water for rinsing post-scrub are necessary to enhance its efficacy. It is important to acknowledge that some resistance might emerge since transitioning from established practice can be difficult. The habit of using traditional methods may pose a challenge to compliance.

### Limitations and future research

A notable limitation of this study is the relatively small sample size, which may limit the generalizability of the results. Additionally, the study did not assess the long-term effects of repeated use of these antiseptic solutions on skin health. Future research should explore the long-term impact of these solutions on hand hygiene and the potential development of bacterial resistance. Furthermore, comparative studies involving larger and more diverse populations are needed to validate these findings and inform clinical guidelines.

## Conclusion

This study underscores the importance of selecting effective antiseptic solutions for hand hygiene in surgical settings. Although both alcohol-based Septicidine and iodine-based Betadine significantly reduced bacterial contamination with no statistically significant difference between them, neither completely eliminated bacteria from the hands of surgical staff. The persistence of antibiotic-resistant strains after scrubbing highlights the need for continuous monitoring and optimization of hand hygiene practices. Considering the advantages of alcohol-based solutions, they should be prioritized in surgical environments, while ongoing research is needed to further enhance their efficacy and safety.

## Supporting information

S1 File Minimal data set. (XLSX)
